# Risk factors for pulmonary infection in patients with non-small cell lung cancer: a Meta-analysis

**DOI:** 10.1186/s12890-024-03171-x

**Published:** 2024-07-22

**Authors:** Jin Chen, Yu Liu, Hong Cai, Wenfa Zheng

**Affiliations:** 1 Department of Oncology, Quanzhou hospital of traditional Chinese medicine, No.388, Sunjiang Road, Jinlong Subdistrict, Licheng District, Quanzhou City, Fujian Province China; 2 Quanzhou Medical Research Institute, Licheng District, Quanzhou City, Fujian Province China

**Keywords:** Non-small cell lung cancer, Pulmonary infection, Meta-analysis

## Abstract

**Background and objectives:**

The aim of this study is to assess and examine the risk variables associated with pulmonary infections in non-small cell lung cancer (NSCLC) and to offer evidence-based recommendations for clinical prophylaxis.

**Methods:**

Up to December 2023, database such as CNKI, Wanfang, VIP Chinese Science and Technology Journals, Chinese Biomedical, Pubmed, Web of science, and the Cochrane library were searched in order to find randomized controlled trials pertaining to non-small cell lung cancer complicated by pulmonary infection. The NOS scale was utilized to assess the quality of the included research, and the Q test findings were utilized to ascertain the degree of heterogeneity among the studies.

**Results:**

After retrieving 957 studies in total, 10 literatures were ultimately included for additional analysis. Diabetes mellitus [OR, 2.89; 95% CI: 1.85–4.52; *P* < 0.00001)] hypoalbuminemia, radiotherapy [OR, 0.43; 95% CI: 1.89–4.07; *P* < 0.00001], and surgical duration exceeding 180 min [OR,1.10 (1.10 ~ 5.38); *P* = 0.03] were found to be risk factors for lung infection. Nevertheless, in NSCLC patients, pulmonary infection was not significantly correlated with factors such as age [OR, -0.16 (-0.96 ~ 0.64); *P* = 0.69], sex [OR, 1.08 (0.77 ~ 1.51); *P* = 0.66], smoking [OR, 1.10 (0.61 ~ 2.00); *P* = 0.75], adenocarcinoma [OR,1.10 (0.55 ~ 2.22); *P* = 0.79], OR, 1.08; 95% CI: 0.77–1.51; *P* = 0.66], TNMIII-IV stages [OR, 1.62; 95% CI: 0.96–2.75; *P* = 0.07], and hypertension [1.01(0.76 ~ 1.34); *P* = 0.94].

**Conclusion:**

Diabetes mellitus, radiation therapy, and longer than 180-minute surgeries are risk factors for lung infection in NSCLC patients. The incidence of lung infection can be reduced by quickly identifying these risk factors and putting preventive measures in place.

**Supplementary Information:**

The online version contains supplementary material available at 10.1186/s12890-024-03171-x.

## Introduction

The most common histological type of lung cancer is non-small cell lung cancer (NSCLC), which accounts for around 75% of all occurrences of lung cancer [1, 2], Because of the country’s accelerating aging population, NSCLC is becoming more common in China, which puts patients’ physical and mental health at serious risk [3]. Chest pain, coughing, and low-grade fever are common clinical symptoms that might result in respiratory and circulatory system deficits, as well as potentially fatal conditions [4, 5]. Patients endure protracted periods of physical exhaustion after the illness, especially the elderly who frequently have coexisting chronic illnesses. As a result, they are less able to fight against many illnesses, especially lung infections. One of the main causes of the poor prognosis linked to this illness is this compromised immune response [6]. In order to lower the frequency of lung infection and improve patient prognoses, it is crucial to identify the risk factors for lung infection in NSCLC patients, recognize them early, and develop logical and efficient preventive and treatment techniques. [7, 8].

Scholars from around the world have shown great interest in the risk factors associated with pulmonary infections in lung cancer patients in recent years, leading to numerous clinical studies being conducted. These studies, however, have incorporated various risk factors and produced inconsistent results. The majority of these have concentrated on lung cancer patients who have pulmonary infections; the risk factors unique to lung cancer that are exacerbated by pulmonary infections have received less attention. Therefore, the purpose of this study is to statistically examine published literature on the risk factors of lung infections in NSCLC patients by using a meta-analysis approach. It is anticipated that the results will offer significant support for clinical preventive tactics.

## Materials and methods

### Sources of materials and retrieval strategies

CNKI, Wanfang, VIP Chinese Science and Technology Journals, Chinese Biomedical, Pubmed, Web of science, Cochrane library, among others were among the databases used for this study. The time frame covered by the search was each database’s creation date until December 2023. The search terms employed in Chinese were “non-small cell lung cancer,” “lung infection,” “risk factors,” “high risk factors,” and so on. The English search keywords, with expanded synonyms, comprised of “non-small cell Lung Cancer,” “Lung Infection,” “Risk Factors,” “High Risk Factors.” The search terms were interconnected with the operator “AND.”

### Inclusion and exclusion criteria

Literature inclusion criteria: (1) The studies that meet the eligibility requirements should be cross-sectional, case-control, and cohort studies conducted in both Chinese and English and published between the start of each database and December 1, 2023. (2) Patients, irrespective of their geography or ethnic background, who have been clinically and pathologically diagnosed with lung cancer, both small cell and non-small cell lung cancer. (3) Individual, tumor-related, complications-related, and other factors linked to lung infection in non-small cell lung cancer are among the exposure factors. (4) The experimental group’s experience with lung infection and the control group’s lack of it are the outcome measures. The study should use diagnostic criteria including blood work, sputum culture, temperature, CT, X-ray, and blood testing. (5) The sample of data duplication by the same author, the study with the largest sample size or the most recently published should be selected.

Exclusion criteria for the literature review encompassed the following factors: (1) duplication studies or reviews that had nothing to do with the topic; (2) absence of randomized controlled trials; (3) lack of clear diagnostic or efficacy criteria; (4) test and control groups that did not have lung infection; (5) data with missing, incomplete, or blatantly erroneous information.

### Literature screening and data extraction

A thorough evaluation of the literature was conducted by two researchers from the research team, using predetermined criteria for inclusion and exclusion. They looked at the abstracts and titles first, then when needed, they retrieved the entire content. When disagreements emerged, they looked to outside experts for advice on how to settle arguments. The literature that met the inclusion criteria was meticulously extracted using a previously established table outlining the characteristics of the literature. Relevant information such as study design type, total sample size, sample size of the test and control groups, as well as outcome indicators, were carefully recorded.

### Literature quality evaluation

Cohort studies literature was evaluated using the Newcastle-Ottawa Scale (NOS) [9]. The NOS comprises three dimensions: selection of study participants, comparability between groups, and assessment of outcomes. It consists of eight items, and a maximum score of 9 points can be obtained. A literature with a score ranging from 5 to 9 points can be considered of high quality.

### Statistical methods

Note Express 3.2 was used for literature management, and Excel 2003 was used for data extraction and collecting from the literature. Software called Revman 5.4.1 was used to do the meta-analysis. The retrieved data was subjected to an I2 value combination and Q test (P value) analysis in order to evaluate heterogeneity. If either *P* > 0.10 or I2 ≤ 50% was found to indicate heterogeneity, the fixed effect model was applied. Forest plots were also used to illustrate the odds ratio (OR) and its associated 95% confidence interval (CI), which were used to convey the results of the pooled study. Funnel plots were used to evaluate publication bias. The two-sided test level is set at α = 0.05.

## Results

### Literature search results

**Fig. 1:**
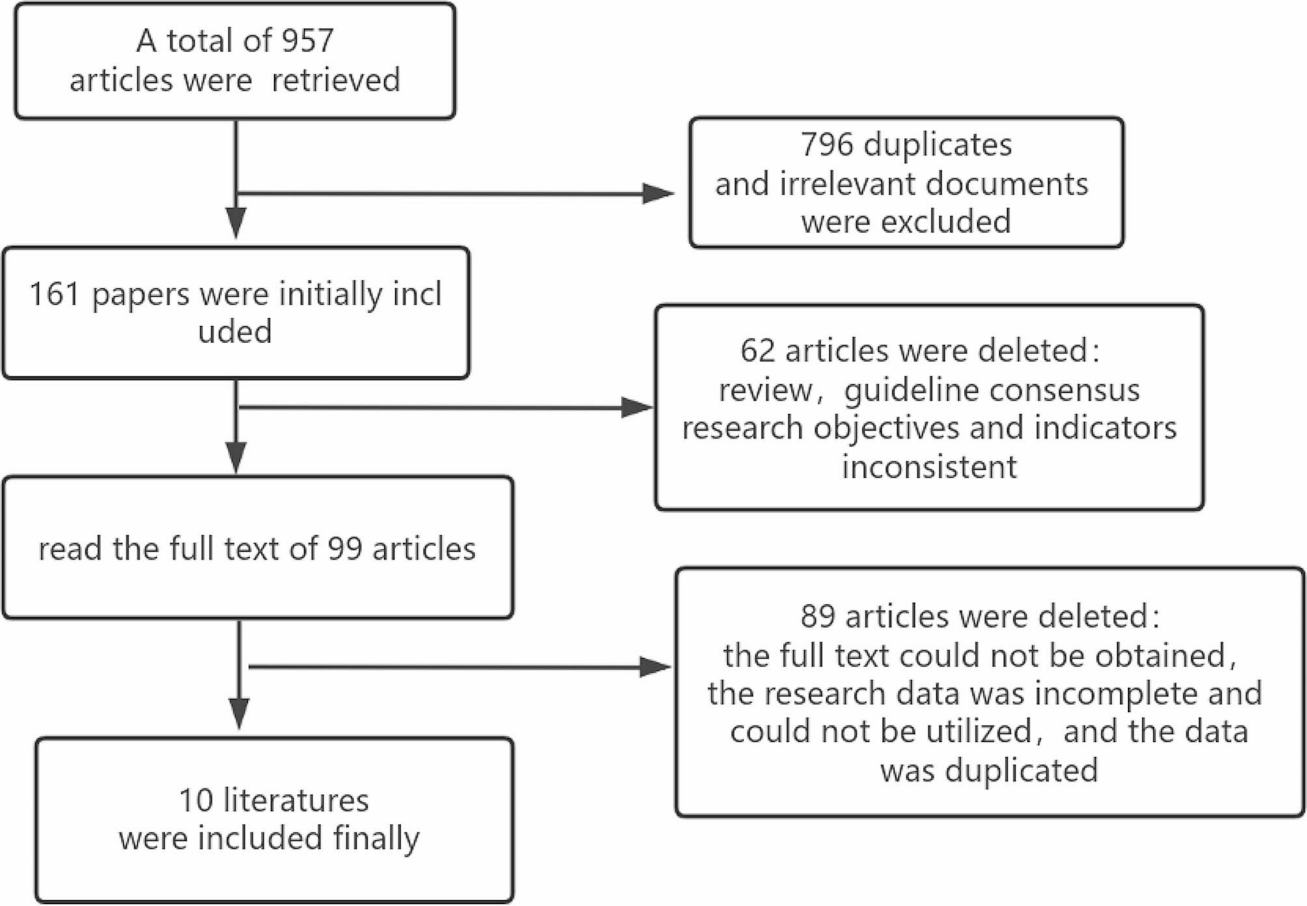
Flow chart of literature screening

A total of 957 pertinent publications were obtained, including China Knowledge Net, Wanfang Database, VIP Chinese Science and Technology Journal Database, China Biomedical Database, Pubmed, Webofscience, Cochanelibrary, among others. Following the removal of duplicate publications across multiple databases, a thorough examination of the title, abstract, and full text ultimately resulted in the inclusion of 10 publications. The literature screening process is illustrated in Fig. 1.

### Basic characteristics and quality evaluation of literature

The baseline data included a range of variables, including age, gender, disease course, treatment plan, and outcome measures. The ten chosen papers offered a thorough summary of the baseline data. A comprehensive overview of the baseline data was provided in the 10 selected studies. To assess the quality, the NOS table was utilized, resulting in 8 studies receiving a score of 5 points and 2 studies receiving a score of 6 points. Please refer to Table 1 for detailed information.

**Table 1:** Basic characteristics and quality evaluation table of literature

first author	year of publication	Sample size (cases)		Exposure factors	NOS score
		test group	control group		
Zhang YX [20]	2019	65	76	1.2.3.4.5	5
Li XC [21]	2022	136	609	2.4.5.6.7.9	6
He L [22]	2020	45	103	2.3.5.6.7.8.10	5
Xu LR [23]	2022	45	66	1.2.4.5	5
Chi RF [24]	2021	45	180	2.3.5.9	5
Wang Q [25]	2021	12	108	2.3.6.7	5
Sun J [26]	2023	62	238	2.8.10	5
Yao N [27]	2023	50	118	2.6.7.8	5
Sun RH [28]	2023	64	120	1.2.3.4.5.6.7	5
Ding Z Z [29]	2023	27	217	2.4.5.6.7.9.10	6

*Note* (1) Age, (2) Gender, (3) Smoking, (4) Adenocarcinoma, (5) TNMIII-IV, (6) Diabetes, (7) Hypertension, (8) Radiotherapy, (9) Duration of surgery ≥ 180 min, (10) Hypoproteinemia

### Meta analysis results

#### Individual factors

**Fig. 2:**
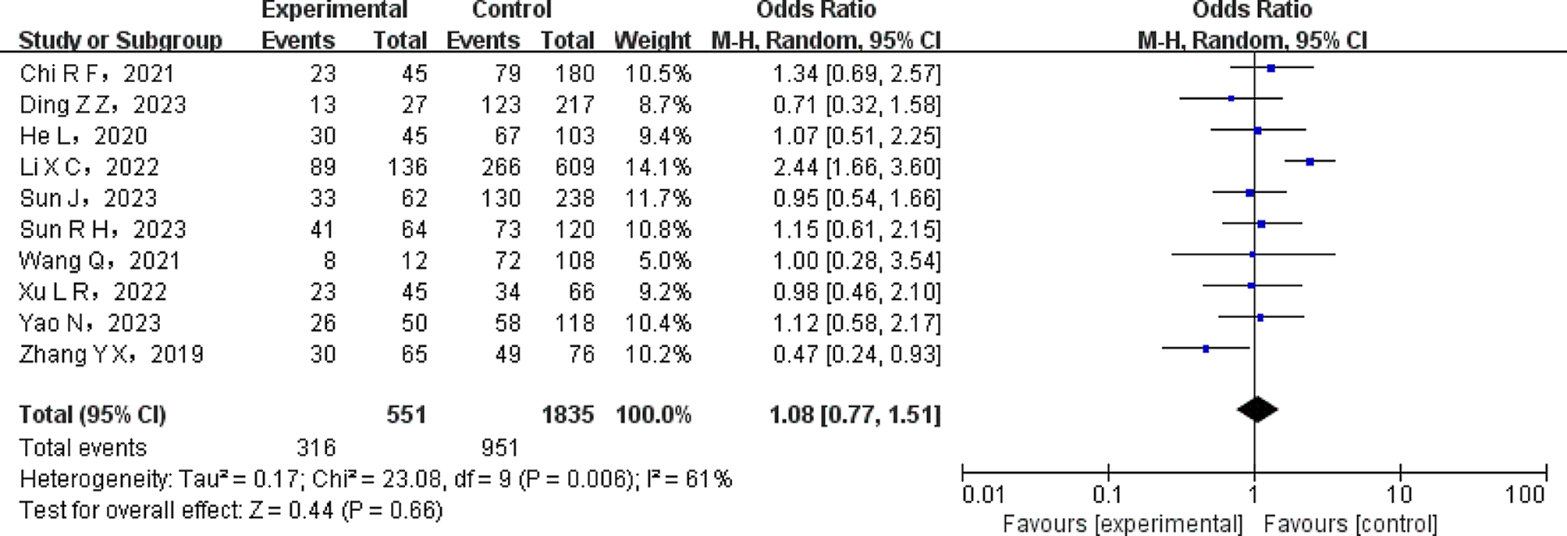
Forest plot of gender factors in NSCLC patients complicated with pulmonary infection. The gender of NSCLC patients complicated with pulmonary shows no significant difference between two groups (REM model, OR, 1.08; 95% CI: 0.77–1.51; *P* = 0.66)

To investigate individual exposure characteristics, such as age, sex, and smoking history, a meta-analysis was carried out. A heterogeneity test was performed on the included literature. The results showed that the age factor did not significantly differ among studies (*P* > 0.1). Consequently, the fixed-effects model (FEM) was used to incorporate the data from the literature. However, there was a significant variation between the studies for the characteristics related to sex and smoking history (*P* < 0.1). Therefore, the pooled data from the literature was analyzed using the random-effects model (REM). Table 2 presents the meta-analysis results, which showed that smoking, age, and gender, as well as other individual characteristics, did not significantly correlate with lung infection in patients with non-small cell lung cancer (all *P* > 0.05). Figure 2 depicts a forest plot illustrating the association between gender factors and lung infection in NSCLC patients.

**Table 2:** Meta-analysis results of individual factors of lung infection in patients with NSCLC

exposure factors	number of literatures	number of patients	heterogeneity test		model selection	OR(95% CI)	*P*
			I2(%)	*P*			
Age	3	436	0	0.89	FEM	-0.16(-0.96 ~ 0.64)	0.69
Sexual	10	2386	61	0.006	REM	1.08(0.77 ~ 1.51)	0.66
Smoking	5	1088	65	0.02	REM	1.10(0.61 ~ 2.00)	0.75

#### Comorbidities

**Fig. 3:**
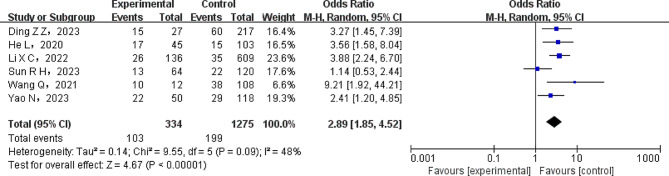
Forest diagram of lung infection in patients with non-small cell lung cancer with or without diabetes. The NSCLC patients with non-small cell lung cancer with or without diabetes shows significant difference between two groups (REM model, OR, 2.89; 95% CI: 1.85–4.52; *P* < 0.00001)

The comorbidities of patients with NSCLC, such as diabetes mellitus, hypertension, and hypoalbuminemia, were investigated by a meta-analysis. A heterogeneity test was run to evaluate the heterogeneity between the included studies. Significant heterogeneity was found amongst the studies regarding diabetes mellitus (*P* < 0.1). Consequently, the data from the literature was combined using the REM. Conversely, no noteworthy heterogeneity was noted in the studies pertaining to hypoalbuminemia and hypertension (*P* > 0.1). Consequently, the pooled data was analyzed using the FEM. According to the meta-analysis findings, individuals with non-small cell lung cancer (NSCLC) were found to have a higher risk of lung infection when they had diabetes mellitus and hypoalbuminemia (*P* < 0.05). Kindly consult Table 3 for further information. As an example, Fig. 3 presents a forest plot focusing on the relationship between diabetes mellitus and lung infection in NSCLC patients.

**Table 3:** Meta-analysis results of individual factors of lung infection in patients with NSCLC

exposure factors	number of literatures	number of patients	heterogeneity test		model selection	OR(95% CI)	*P*
			I2(%)	*P*			
Diabetes	6	1609	48	0.09	REM	2.89(1.85 ~ 4.52)	< 0.00001
Hypertension	6	1798	0	0.76	FEM	1.01(0.76 ~ 1.34)	0.94
Hypoalbuminemia	3	692	0	0.41	FEM	4.00(2.68 ~ 5.97)	< 0.00001

#### Tumor factors

**Fig. 4:**
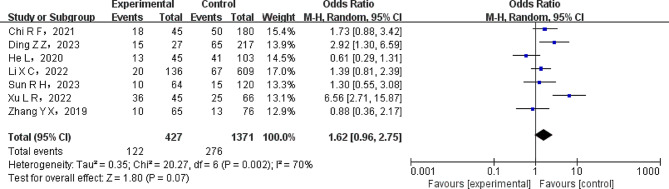
Forest map of TNM staging of lung infection in patients with NSCLC. The NSCLC patients with TNM staging shows no significant difference between two groups (REM model, OR, 1.62; 95% CI: 0.96–2.75; *P* = 0.07)

A meta-analysis was conducted to assess the relationship between lung cancer subtype (adenocarcinoma) and tumor-node-metastasis (TNM) stage (stage III ~ IV), among other tumor factors. Heterogeneity tests were performed on the included studies, revealing statistically significant heterogeneity (*P* < 0.1). To incorporate the data from the literature for a combined analysis, a REM was used. Table 4 displays the results of the meta-analysis, which showed that in patients with NSCLC, there was no significant correlation (all *P* > 0.05) between adenocarcinoma, TNM stage III–IV, and other variables with lung infection, as presented in Table 4. Figure 4 presents a forest plot depicting the relationship between lung infection and NSCLC patients stratified by TNM stage.

**Table 4:** Meta-analysis results of individual factors of NSCLC patients complicated with pulmonary infection

exposure factors	number of literatures	number of patients	heterogeneity test		model selection	OR(95% CI)	*P*
			I2(%)	*P*			
Adenocarcinoma	5	1045	81	0.0004	REM	1.10(0.55 ~ 2.22)	0.79
TNM III ~ IV Stages	7	1798	70	0.002	REM	1.62(0.96 ~ 2.75)	0.07

#### Tumor treatment factors

**Fig. 5:**
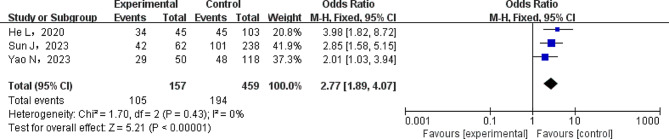
Forest diagram of NSCLC patients complicated with lung infection after radiotherapy. The NSCLC patients with after radiotherapy shows significant difference between two groups (FEM model, OR, 0.43; 95% CI: 1.89–4.07; *P* < 0.00001)

To investigate the impact of radiation therapy and length of operation on tumor treatment results, a meta-analysis was carried out. A heterogeneity test was performed on the included literature. When radiotherapy was taken into account as the exposure factor, the results showed that there was no significant variance among the studies (*P* > 0.1). Therefore, the literature data were combined using a fixed-effects model (FEM). Nonetheless, there was a significant amount of heterogeneity among the literature studies when the operation duration was more than 180 min (*P* < 0.1). It was therefore decided to perform a pooled analysis of the literature data using a random-effects model (REM). The meta-analysis’s findings showed a strong correlation (all *P* < 0.05) between lung infection and tumor treatment factors (such radiation and surgeries lasting more than 180 min) in patients with non-small cell lung cancer. For further details, please see Table 5. A forest plot is shown in Fig. 5 to show how lung infection and radiation therapy relate to each other in patients with non-small cell lung cancer.

**Table 5:** Meta-analysis results of individual factors of NSCLC patients complicated with pulmonary infection

exposure factors	number of literatures	number of patients	heterogeneity test		model selection	OR(95% CI)	*P*
			I2(%)	*P*			
Radiotherapy	3	616	0	0.43	FEM	2.77(1.89 ~ 4.07)	< 0.00001
surgical duration exceeding 180 min	3	1214	71	0.03	REM	1.10(1.10 ~ 5.38)	0.03

### Bias check for literature

**Fig. 6:**
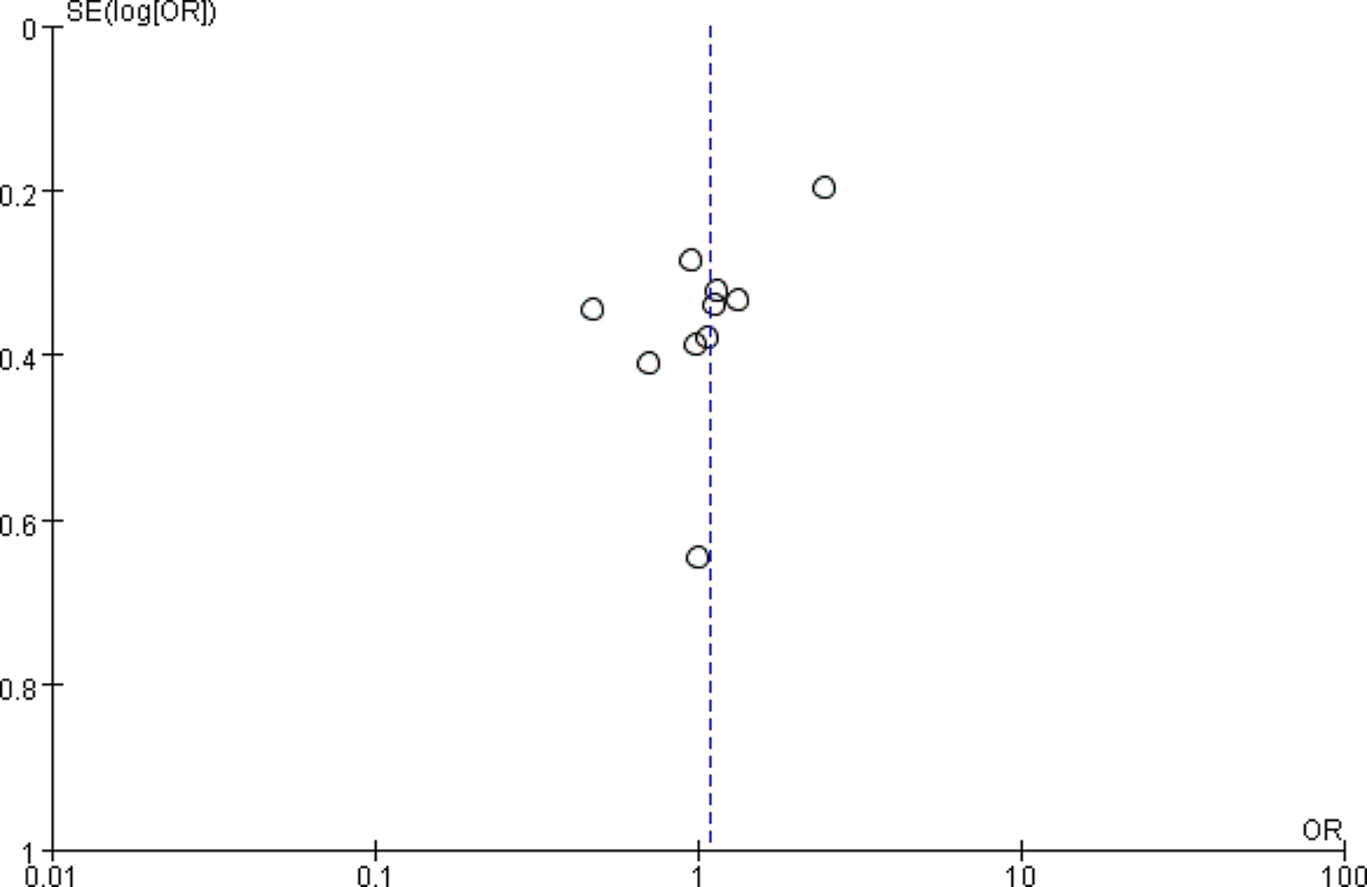
Funnel plot with gender as an example

Bias check for all outcome indicators involved in this article showed asymmetry on both sides, suggesting bias. Take gender as an example, see Fig. 6.

## Discussion

The majority of cases of lung cancer are mostly NSCLC. Because of its modest symptoms, it is frequently difficult to identify in its early stages, which results in an advanced diagnosis. As a result, many times the ideal window for major surgery is passed [10–12]. Patients with non-small cell lung cancer (NSCLC) have weakened immune systems, reduced resistance, and bone marrow hematopoietic suppression. These features make them more vulnerable to lung infections. [13, 14].

According to this meta-analysis, there was a significant correlation between the incidence of lung infection in patients with non-small cell lung cancer (NSCLC) and diabetes mellitus [OR, 2.89; 95% CI: 1.85–4.52; *P* < 0.00001], hypoalbuminemia [OR,4.00; 95% CI:2.68 ~ 5.97; *P* < 0.00001], administration of radiotherapy [OR, 0.43; 95% CI: 1.89–4.07; *P* < 0.00001], and surgical duration exceeding 180 min[OR,1.10; 95% CI:1.10 ~ 5.38; *P* = 0.03] (*P* ≤ 0.05). This connection could be attributed to several variables. Firstly, a prolonged high glucose environment brought on by diabetes mellitus encourages the growth of microorganisms. It is suggested that hyperglycemia impairs the function of lung dendritic cells, which are crucial for activating the adaptive immune response against viral infections [15]. Furthermore, there is a greater chance of tissue hypoxia and reduced tissue perfusion due to poor phagocytic function, neutrophil chemotaxis, and insufficient blood vessel supply, which raises the risk of lung infection. Consequently, blood sugar should be carefully managed and lung infection should be decreased in individuals with combination diabetes [16, 17]. Secondly, hypoalbuminemia (low serum albumin levels) is associated with an increased risk and severity of infectious diseases. This is because intact innate and adaptive immune responses depend on albumin, making individuals more vulnerable to lung infection [18]. Thirdly, vascular thickening and blockage brought on by radiation therapy, which is frequently administered to elderly NSCLC patients, can hinder normal tracheal mobility. Consequently, this diminishes the tracheal self-purification ability and contributes to radiation pneumonia in patients [19]. Therefore, optimizing blood glucose control and nutritional status are important interventions to mitigate the risk of lung infections in this patient population.

There are several constraints associated with this research: the meta-analysis revealed a bias, potentially attributed to the extensive time frame and inadequate sample size of the literature incorporated in this study. Furthermore, only Chinese and English databases were utilized for retrieval, and the selective inclusion of literature in each database resulted in a potential sampling bias that may have influenced the study’s findings.

## Conclusion

In summary, the risk of lung infection in patients with non-small cell lung cancer (NSCLC) is independently increased by diabetes mellitus, radiation therapy, and operations lasting longer than 180 min. The incidence of lung infections in patients can be reduced with early detection and the application of preventive measures.

### Electronic supplementary material

Below is the link to the electronic supplementary material.

Supplementary Material 1

Supplementary Material 2

## Data Availability

The data analyzed in the present manuscript can be provided under reasonable requests.

## Declarations

### Clinical trial number

The present study is a meta-analysis and literature review, therefore clinical trial number is not applicable.
