# Supplementary material for: Risk factors for pulmonary infection in patients with non-small cell lung cancer: a Meta-analysis
Source: BMC Pulm Med. 2024 Jul 22;24:353. doi: 10.1186/s12890-024-03171-x (PMC11265114; doi:10.1186/s12890-024-03171-x)
Supplement: Supplementary file 2 — Supplementary Material 2 [file 12890_2024_3171_MOESM2_ESM.docx]

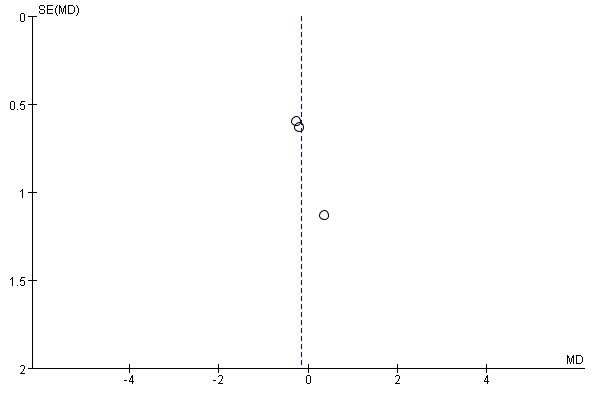


Fig. 1. Funnel plot with age


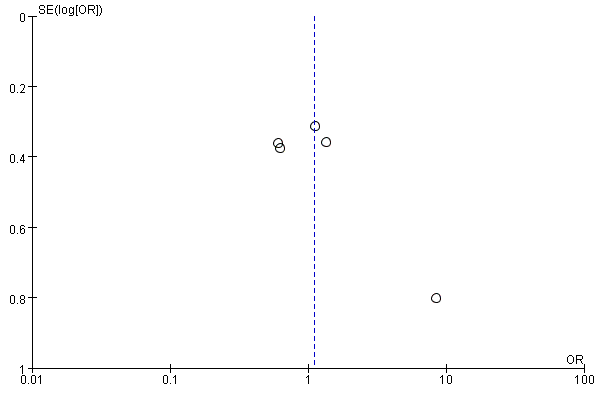


Fig. 2. Funnel plot with smoking


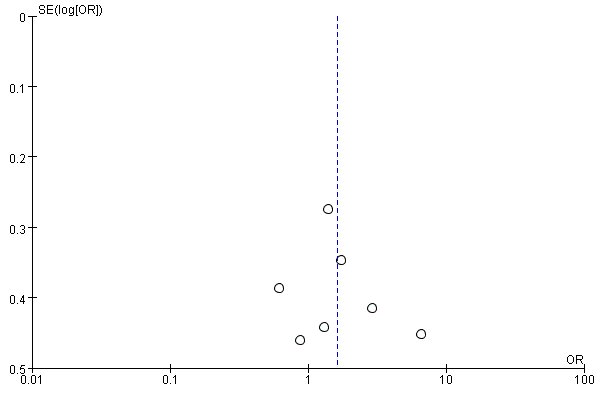


Fig.3. Funnel plot with TNM staging


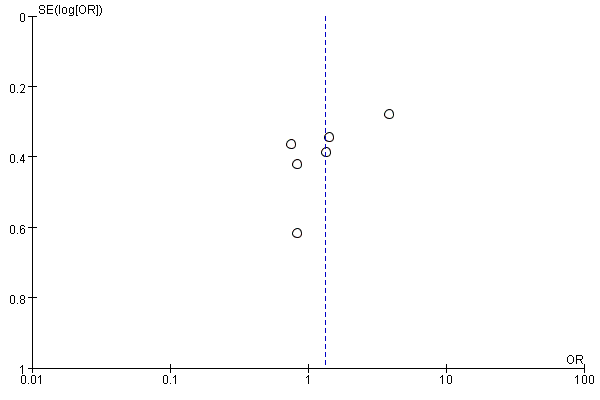


Fig.4. Funnel plot with TNM hypertension


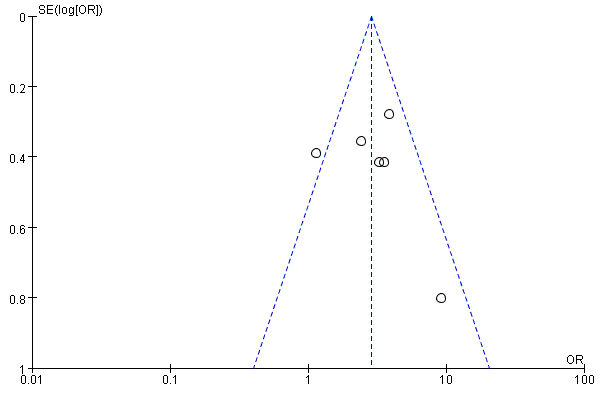


Fig.5. Funnel plot with diabetes


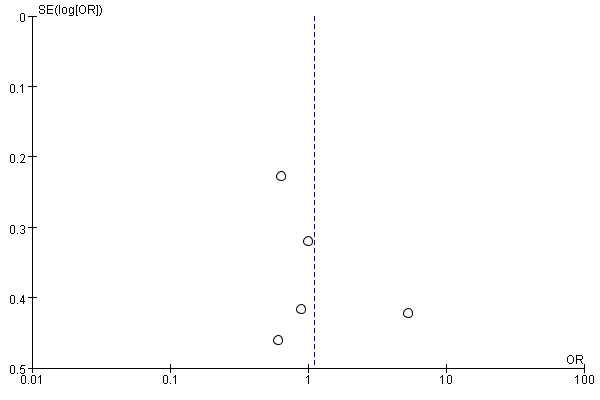


Fig.5. Funnel plot with adenocarcinoma
